# Heterologous Production of an Energy-Conserving Carbon Monoxide Dehydrogenase Complex in the Hyperthermophile *Pyrococcus furiosus*

**DOI:** 10.3389/fmicb.2016.00029

**Published:** 2016-01-29

**Authors:** Gerrit J. Schut, Gina L. Lipscomb, Diep M. N. Nguyen, Robert M. Kelly, Michael W. W. Adams

**Affiliations:** ^1^Department of Biochemistry and Molecular Biology, University of Georgia, AthensGA, USA; ^2^Department of Chemical and Biomolecular Engineering, North Carolina State University, RaleighNC, USA

**Keywords:** hyperthermophile, archaea, hydrogen, carbon monoxide, energy, anaerobic respiration, Thermococcales

## Abstract

Carbon monoxide (CO) is an important intermediate in anaerobic carbon fixation pathways in acetogenesis and methanogenesis. In addition, some anaerobes can utilize CO as an energy source. In the hyperthermophilic archaeon *Thermococcus onnurineus*, which grows optimally at 80°C, CO oxidation and energy conservation is accomplished by a respiratory complex encoded by a 16-gene cluster containing a CO dehydrogenase, a membrane-bound [NiFe]-hydrogenase and a Na^+^/H^+^ antiporter module. This complex oxidizes CO, evolves CO_2_ and H_2_, and generates a Na^+^ motive force that is used to conserve energy by a Na^+^-dependent ATP synthase. Herein we used a bacterial artificial chromosome to insert the 13.2 kb gene cluster encoding the CO-oxidizing respiratory complex of *T. onnurineus* into the genome of the heterotrophic archaeon, *Pyrococcus furiosus*, which grows optimally at 100°C. *P. furiosus* is normally unable to utilize CO, however, the recombinant strain readily oxidized CO and generated H_2_ at 80°C. Moreover, CO also served as an energy source and allowed the *P. furiosus* strain to grow with a limiting concentration of sugar or with peptides as the carbon source. Moreover, CO oxidation by *P. furiosus* was also coupled to the re-utilization, presumably for biosynthesis, of acetate generated by fermentation. The functional transfer of CO utilization between *Thermococcus* and *Pyrococcus* species demonstrated herein is representative of the horizontal gene transfer of an environmentally relevant metabolic capability. The transfer of CO utilizing, hydrogen-producing genetic modules also has applications for biohydrogen production and a CO-based industrial platform for various thermophilic organisms.

## Introduction

Carbon monoxide (CO) is a high energy compound with a very low reduction potential for the CO/CO_2_ couple [*E_0_′* = -524 mV: (Thauer, 1990)], see Equation 1.

(1) $$\text{CO}\;\text{+}\;{\text{H}}_{2}\text{O}\;\to \;{\text{CO}}_{2}\;\text{+}\;{\text{2H}}^{+}\;\text{+}\;{\text{2e}}^{-}$$

Anaerobic microorganisms that directly use CO as a carbon source do so via the Wood-Ljungdahl pathway. This is present in acetogenic bacteria and methanogenic archaea (Wood, 1991; Costa and Leigh, 2014) and has been studied for more than 80 years (Drake and Daniel, 2004). The key enzyme is a bifunctional and bimodular CO dehydrogenase/acetyl-CoA synthase (Codh/Acs) complex. This enzyme combines CO with a methyl group, donated by a corrinoid containing carrier protein, with coenzyme A to generate acetyl-CoA (Ragsdale et al., 1996). The Wood–Ljungdahl pathway functions to fix CO_2_ into the cellular material via acetyl-CoA where hydrogen gas is commonly used as the source of reductant (Ragsdale et al., 1996). The pathway consists of two branches. One reduces CO_2_ to the level of the methyl group in acetyl-CoA while the other reduces CO_2_ to CO, which forms the carbonyl group of acetyl-CoA (Schuchmann and Muller, 2014).

A diverse group of microorganism are able to use CO as a sole energy source independent of the Wood–Ljungdahl pathway (Oelgeschlager and Rother, 2008). Some anaerobes can utilize CO in the absence of an electron acceptor by coupling CO oxidation to the reduction of protons (H_2_/H^+^, *E_0_′* = -414 mV, pH 7.0) thereby releasing a significant amount of energy (*ΔG^0^’* = -27 kJ/mol). The thermophilic Firmicute *Carboxydothermus hydrogenoformans* grows very well using CO as a sole source of both energy and carbon (Uffen, 1976; Svetlichny et al., 1991; Wu et al., 2005) and has become the model system for studying CO-dependent growth by biochemical, physiological and evolutionary approaches (Soboh et al., 2002; Henstra and Stams, 2011; Techtmann et al., 2012). The membrane-bound H_2_-evolving Codh complex of *C. hydrogenoformans* contains eight subunits. Two of these represent the Codh module while the other six are homologous to the energy converting hydrogenase (Ech) first characterized in the methanogen *Methanosarcina barkeri* (Meuer et al., 1999). Hence the *C. hydrogenoformans* Codh complex is a bimodular system in which electrons from CO oxidation are directed to an ion-translocating hydrogenase (Svetlitchnyi et al., 2001; Wu et al., 2005). The Codh module of *C. hydrogenoformans* appears to be similar to the Codh module of the Codh/Acs complex found in acetogens and methanogens and contains a similar [Ni-4Fe-5S] cluster as part of its catalytic site (Dobbek et al., 2001; Techtmann et al., 2012).

The CO utilization pathways of acetogenic, methanogenic, and hydrogenogenic carboxydotrophs exemplify how CO can be metabolized under anaerobic conditions without an external electron acceptor (besides CO_2_). In such cases acetogens generate CO_2_ and acetate, methanogens produce CO_2_ and CH_4_, and the hydrogenotrophic carboxydotrophs convert CO to CO_2_ and H_2_ (Oelgeschlager and Rother, 2008; Sokolova et al., 2009). It should be noted that *C. hydrogenoformans* is an autotroph and uses the Wood–Ljungdahl pathway for carbon fixation. Thus this organism contains a bimodular Codh/Acs complex in addition to a bimodular Codh/Ech complex (Svetlitchnyi et al., 2004; Henstra and Stams, 2011).

The heterotrophic hyperthermophilic archaeon *Thermococcus onnurineus* grows optimally at 80°C and was recently shown to gain energy for growth by coupling CO oxidation to H_2_ production (Rittmann et al., 2015). *T. onnurineus* has been extensively used as a model organism for the production of biohydrogen from various one-carbon compounds such as formate and CO. However, *T. onnurineus* is not an autotroph and requires a source of organic carbon in the form of peptides for growth on CO (Bae et al., 2012). *T. onnurineus* is a member of the archaeal order Thermococcales, members of which grow optimally in the temperature range from 75 to 100°C (Schut et al., 2014). A hallmark of this group is the presence of a respiratory membrane-bound hydrogenase complex (MBH) that is responsible for evolving H_2_ as a means for disposing of the reductant generated from fermentation. MBH consists of two modules. One is a five-subunit respiratory hydrogenase (Mbh) that evolves H_2_ and generates a proton gradient (Sapra et al., 2003). The other is a nine-subunit Na^+^/H^+^ antiporter (Mrp) that converts the proton gradient into a Na^+^ ion gradient. How exactly the Na^+^ gradient is made is not clear; however, in a homologous formate-dependent system it was proposed that a proton gradient is formed first and is then converted to a Na^+^ gradient (Lim et al., 2014). It is clear that ultimately a Na^+^ gradient needs to be established in order for energy to be conserved by a Na^+^-dependent ATP synthase (Pisa et al., 2007).

**FIGURE 1:**
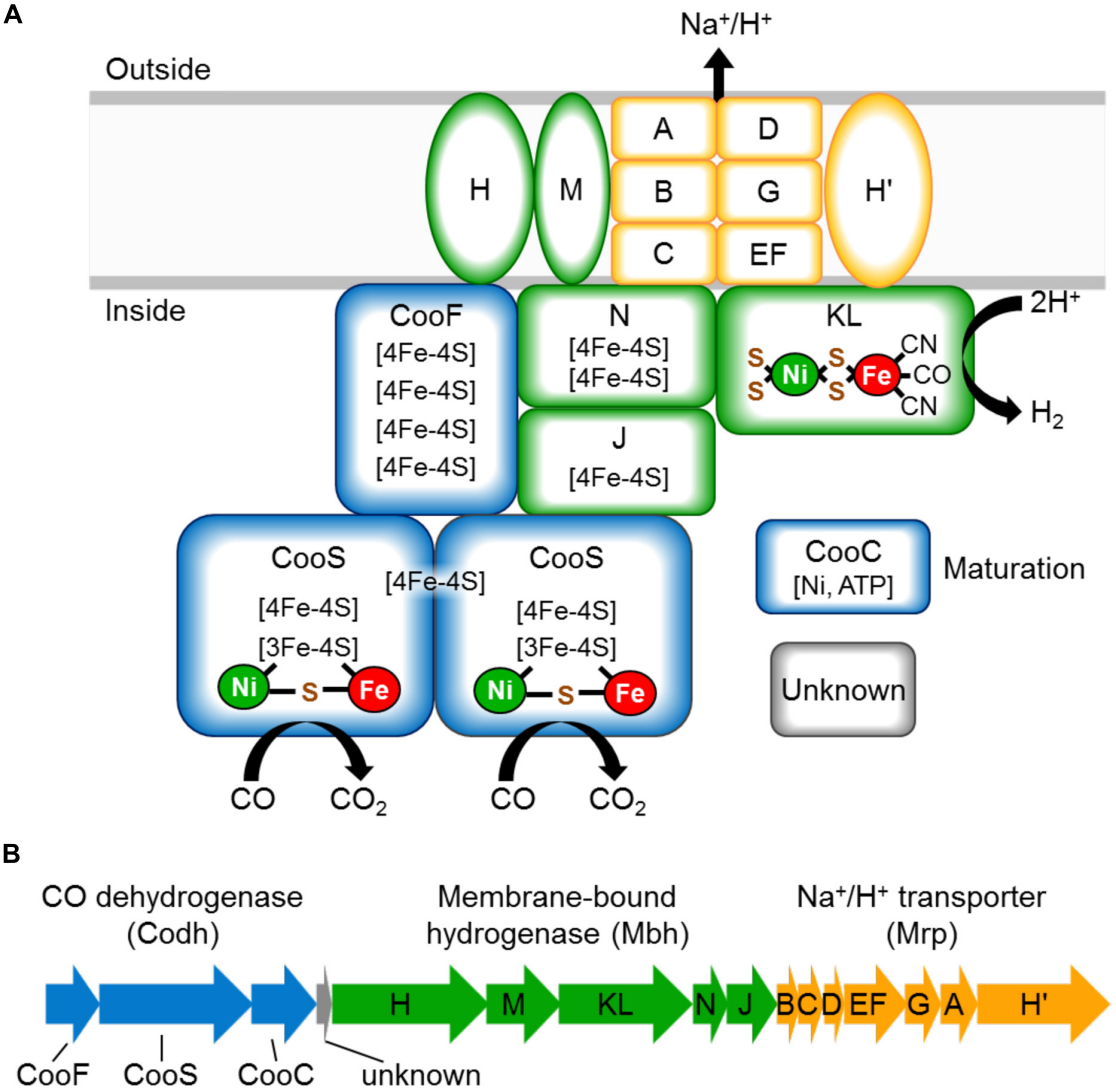
**(A)** Schematic representation of the CO oxidizing Codh complex of *Thermococcus onnurineus*, which conserves energy through an ion gradient. The naming of the Mrp and Mbh subunits is matched to those of the Mrp and Mbh subunits of *Pyrococcus furiosu*s (Schut et al., 2013). **(B)** Operon structure of the Mrp-Mbh-Codh encoding genes (TON_1017-TON_1031). The gene encoding subunit *N* is depicted here as we have sequence verified that there is no frameshift in this gene, contrary to the published genome sequence.

The CO-oxidizing Codh complex of *T. onnurineus* is therefore trimodular in nature (Mrp-Mbh-Codh) (Kim et al., 2013). It consists of a 12-subunit hydrogenase-antiporter complex (Mrp-Mbh) together with the two subunits of CO dehydrogenase with proposed structure shown in **Figure 1A**. By analogy with the known structure of the cytoplasmic CO dehydrogenase of acetogens (Kung et al., 2009), the Codh module of the *T. onnurineus* complex is proposed to contain two CooS subunits and one CooF subunit. *T. onnurineus* contains a 16-gene putative operon that encodes the complete Mrp-Mbh-Codh complex (**Figure 1B**) (Lim et al., 2010). This also includes two genes encoding a maturation factor for the Codh module and a protein with unknown function.

The heterotrophic archaeon, *Pyrococcus furiosus* grows in the range of 70–103°C (Fiala and Stetter, 1986) and is also a member of the Thermococcales. Like *T. onnurineus. P. furiosus* is a strict heterotroph and does not fix CO_2_, but, unlike *T. onnurineus*, it cannot utilize CO as a source of energy. *P. furiosus* ferments a range of sugars and uses a respiratory MBH complex (Mrp-Mbh) to dispose of the excess reductant as H_2_ (Sapra et al., 2003; McTernan et al., 2014). It has many desirable features as a metabolic engineering host (Lipscomb et al., 2011; Hawkins et al., 2013; Keller et al., 2013). In this study, we engineered *P. furiosus* to produce the entire *T. onnurineus* Codh complex (Mrp-Mbh-Codh) and show that the resulting strain not only oxidizes CO to produce H_2_, it also grows using CO as a source of energy.

## Materials and Methods

### Strain Construction

The 22.2 kb BAC vector, pGL058 (Basen et al., 2014), containing the Codh gene cluster expression construct for homologous recombination into the *P. furiosus* chromosome at genome region five between convergent genes PF1232 and PF1233 was linearized using the unique *Xho*I restriction site (within *repE* in the BAC vector backbone) and used to transform *P. furiosus* COM1 according to previously published methods (Lipscomb et al., 2011). Transformant colonies were cultured in defined cellobiose (DC) medium (Lipscomb et al., 2011), and gDNA was isolated using the ZymoBead^TM^ Genomic DNA Kit (Zymo Research) for PCR screening. PCR screens were performed using the SpeedSTAR HS DNA polymerase (Takara) for amplification of the ∼16 kb product with primers ∼100 b outside the chromosomal homologous flanking regions. PCR-verified isolates were further purified twice on solid DC medium prior to final PCR screening and saving of glycerol stocks. One of the purified pGL058 transformants, designated MW191, was selected for phenotypic analyses. This strain is referred to herein as the Codh strain.

### Growth and Cell Protein Quantitation

*Pyrococcus furiosus* strains (**Table 1**) were cultured in artificial seawater medium containing per liter: 1× base salts, 1× trace minerals, 1 μM sodium tungstate, 0.25 μg resazurin, 0.5 g cysteine, 0.5 g, 1 g sodium bicarbonate, and 1 mM potassium phosphate buffer, with pH adjusted to 6.8 prior to bottling (Adams et al., 2001). Media was aliquoted into serum bottles (50 mL per 150 mL bottle), and the headspace was replaced with argon after three cycles of vacuum and argon. For growth curves, this medium was supplemented with 1 g L^-1^ yeast extract (Difco) with or without 0.5 g L^-1^ maltose. CO was added as a 100% headspace with a light over pressure (1.2 atm). Medium was inoculated to ∼3 × 10^6^ cells mL^-1^, and cultures were incubated at 80°C with shaking.

**Table 1:** *Pyrococcus furiosus* strains used and constructed in this work.

Strain	Alias	Parent	Genotype/Description	Reference
MW002	COM1^a^	DSM3638^a^	Δ*pyrF*	Lipscomb et al., 2011
MW004	COM1c	MW002	Δ*pyrF*::*pyrF*	Thorgersen et al., 2014
MW191	Codh	MW002	Δ*pyrF* P_gdh_-*pyrF* P_mbh_-(TON1017-1031)	This work

*^a^GenBank genome accession nos.: DSM 3638, AE009950.1; COM1, AE009950.1.*

For extract preparation, growth medium was supplemented with 2 g maltooligosaccharides and 1 g yeast extract per liter, and cultures were incubated at 80°C with stirring. Cell growth was assessed by monitoring total cell protein using a Bradford protein assay kit (Bio-Rad). Briefly, cells were harvested by centrifugation from 1 mL culture samples and lysed by osmotic shock in an equal volume of water, with vortexing and one freeze-thaw cycle. Lysate was centrifuged at 10,000 *g* for 1 min to pellet insoluble cell debris prior to quantitation of soluble cell protein.

### Cell Free Extract Preparation and Enzyme Assays

To obtain cell-free extracts, *P. furiosus* cell pellets were suspended in 50 mM EPPS pH 8.4, with 2 mM sodium dithionite (3 mL buffer per gram of cells) under strict anaerobic conditions. The cells were lysed by osmotic shock and sonication (Qsonica model Q55, 1 min at amplitude 30). In order to prepare membrane extracts, the cell-free extract was centrifuged at 100,000 *g* for 1 h. The resulting pellet was suspended in 8 mL buffer, centrifuged again, and the pellet was suspended in 0.8 mL buffer followed by storage at 4°C in stoppered glass vials until assayed. CO oxidizing:H_2_ formation activity was measured by the production of H_2_ from CO. Glass vials (8 mL volume) fitted with butyl rubber stoppers and containing 1 mL buffer (50 mM EPPS, pH 8.4) with a 100% CO headspace were preheated to 80°C, and the reaction was initiated by the addition of extracts (∼1 mg mL^-1^). Gas samples were analyzed by GC (Shimadzu GC8A with TCD detector, oven 70°C, injector/detector 120°C, Alltech Molecular Sieve column 5A 80/100). Benzyl viologen-dependent Codh activity was measured in 3 mL stoppered glass cuvettes containing 2 mL of anaerobic 50 mM EPPS, pH 8.4, and a 100% CO headspace. Reactions were started after preheating at 75°C with extract (∼2 μg ml^-1^), benzyl viologen reduction was monitored at 600 nm (𝜀 = 7400 M^-1^ cm^-1^).

### H_2_ Quantitation

H_2_ was determined by sampling the headspace of closed bottle cultures with a pressure-lock syringe and analyzing H_2_ content by GC (see above). H_2_ production is expressed as molar amount produced per liquid phase.

### Organic Acid Quantitation

Organic acids were measured using an Agilent 7890A GC equipped with a carbowax/20 m column and an FID detector. Culture samples of 1 mL were centrifuged at 10,000 *g* for 10 min, and the supernatant was acidified with formic acid (100 mM) before analysis.

## Results

### Insertion of the *T. onnurineus* Gene Cluster Encoding the Codh Complex into the *P. furiosus* Chromosome

The complete Codh complex (Mrp-Mbh-Codh) encoded by gene cluster TON_1017-TON_1031 is 13.2 kb in size and is assumed to consist of one transcriptional unit. The published genome sequence lacks the annotation for the N subunit gene (between TON_1023 and TON_1024) due to a sequencing error that introduced a base deletion and frameshift in this gene. This mistake has been reported by Lim et al. (2010), and we also verified the sequence for this work. We depict the correct operon structure including the N subunit gene in **Figure 1**. This TON_1017-TON_1031 gene cluster was inserted into the *P. furiosus* genome under the control of the promoter for the Mbh operon of *P. furiosus* (P*_mbh_*). A bacterial artificial chromosome (BAC) vector was used to facilitate construction of the 15.3 kb genome insertion. This contained 0.5 kb flanking regions for targeted homologous recombination, the P_gdh_-*pyrF* selectable marker cassette, and the Mrp-Mbh-Codh Codh gene cluster expression construct with P*_mbh_*. To avoid potential deleterious effects on the host, the expression construct was inserted into a chromosomal region of low transcriptional activity as determined from analysis of tiling array data (Yoon et al., 2011). The genetically competent strain of *P. furiosus*, termed COM1 (Lipscomb et al., 2011), was transformed with the expression construct containing the Codh gene cluster of *T. onnurineus*, generating the strain MW191, referred to herein as the Codh strain of *P. furiosus*.

### *In Vitro* Evaluation of Codh Complex Activity

To evaluate the activity of the engineered Codh strain of *P. furiosus*, it was grown in the presence of CO at 80°C, near the optimal growth temperature of *T. onnurineus.* Cell-free extracts were prepared for *in vitro* activity measurements. The *T. onnurineus* Codh complex converts CO to equimolar amounts of H_2_ and CO_2_, according to Equation 2.

(2) $$\text{CO}\;\text{+}\;{\text{H}}_{2}\text{O}\;\to \;{\text{CO}}_{2}\;\text{+}\;{\text{H}}_{2}$$

In order to distinguish Codh activity from that of the native MBH activity in *P. furiosus*, Codh activity was measured in *T. onnurineus* cell extracts grown on CO as a control. Cell-free extracts of the *P. furiosus* parental control strain COM1c had no detectable CO-oxidizing activity using either the H_2_ production or benzyl viologen reduction assay. However, cell-free extracts of the *P. furiosus* Codh strain and of *T. onnurineus* had similar CO-dependent H_2_ formation activities (∼0.3 units mg^-1^; **Table 2**). After ultracentrifugation to separate the membrane fraction, this activity remained associated with the membrane, although in both cases more than half of the total activity was lost in the procedure (**Table 2**). As expected, the cytosolic fractions of the two strains did not contain any detectable CO-dependent H_2_ formation activity. However, they did contain high CO-oxidizing activity using benzyl viologen as the electron acceptor, and this represented virtually all (80–97%, **Table 2**) of the CO-oxidizing activity in the cell-free extract. These data suggest that the Codh module of the membrane-bound Codh complex (Mrp-Mbh-Codh) is at best loosely associated with the other two membrane-bound modules (Mrp and Mbh).

**Table 2:** CO-dependent activity in cellular extracts of *P. furiosus* strains Codh and COM1c and in *Thermococcus onnurineus*.

Extract ^a^	CO to H_2_^a^	Recovery	CO to BV ^a^	Recovery
**COM1c control**				
WCE	ND		<0.05	
Cyt	ND		<0.05	
Mem	ND		<0.05	
**Codh strain**				
WCE	0.10 ± 0.03	100%	14.1 ± 4.3	100%
Cyt	ND		13.6 ± 9.3	97%
Mem	0.27 ± 0.11	48%	2.2 ± 0.9	3%
***Thermococcus onnurineus***				
WCE	0.17 ± 0.07	100%	10.0 ± 6.2	100%
Cyt	ND		8.3 ± 4.6	82%
Mem	0.34 ± 0.17	36%	2.0 ± 0.5	4%

*^a^Abbreviations are as follows: WCE, whole cell extract; Cyt, cytoplasmic extract; Mem, membrane fraction; ND, not detected; BV, benzyl viologen. Activity is expressed as μmole CO/min/mg, and recovery represents the total activity obtained after cell fractionation.*

### Growth of *P. furiosus* in the Presence of CO

**FIGURE 2:**
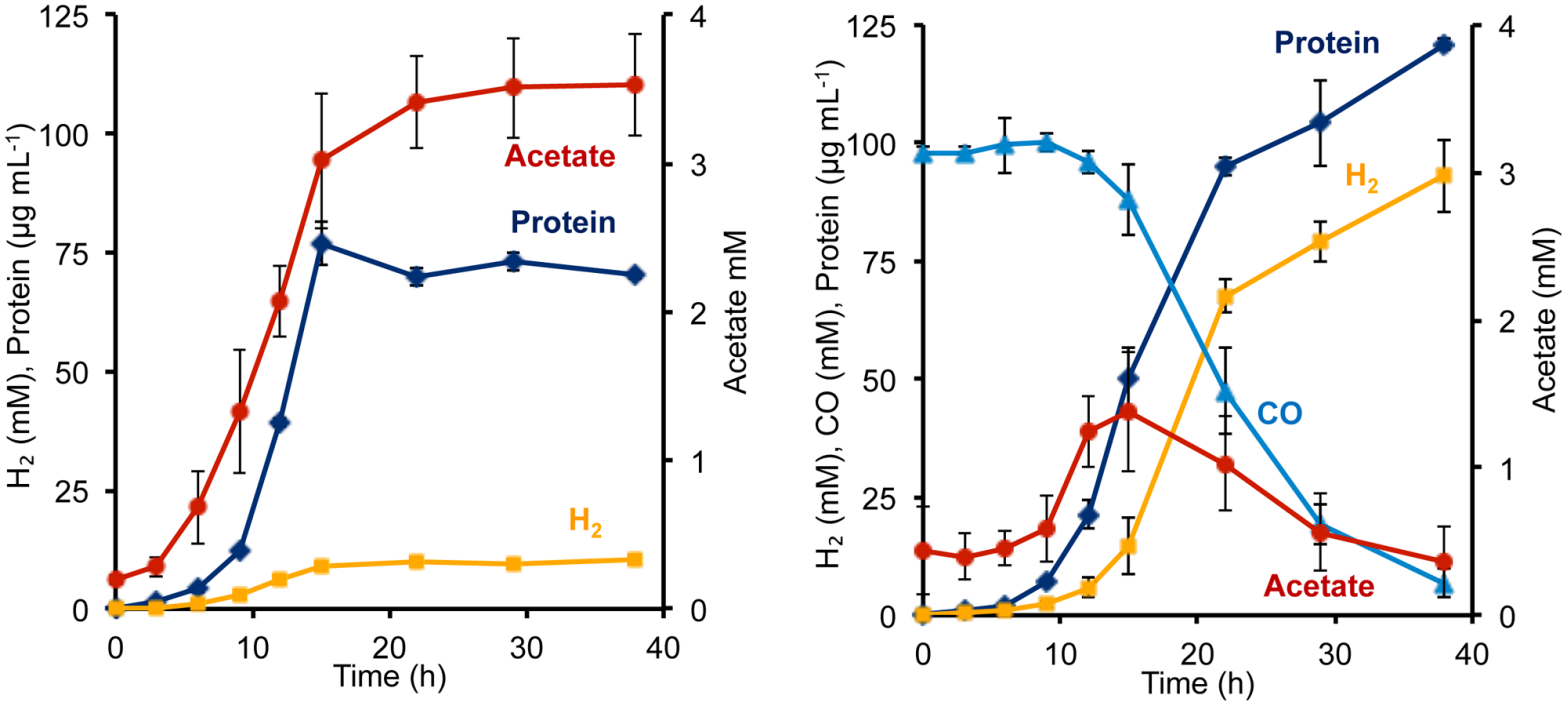
**Growth characteristics of *P. furiosus* strain Codh in the absence **(left)** and presence **(right)** of CO with limiting maltose (0.5 g L^-1^) and yeast extract (1 g L^-1^).** Compounds are represented as follows: blue triangles, CO; dark blue diamonds, cell protein; orange squares, H_2_; red circles, acetate. Error bars represent SD, *n* = 3.

To determine the phenotypic effect of the *T. onnurineus* Codh gene cluster on *P. furiosus*, the Codh strain and the COM1c control strain were grown with and without CO. The presence of 1.2 atm of CO did not seem to inhibit growth of either strain (**Figure 2**, Supplementary Figure S1). To ensure that the *T. onnurineus* Codh complex would be active, strains were grown at 80°C, near the optimal growth temperature of *T. onnurineus*. *T. onnurineus* has been shown to obtain energy for growth from CO oxidation (Yun et al., 2011). Consequently, in order to assess the effect of CO on the *P. furiosus* Codh strain, the amount of available organic carbon was limited in order to maximize the potential growth stimulating effect of CO oxidation. When a limiting amount of maltose was added as the carbon source, the *P. furiosus* Codh strain grew well, reaching ∼75 mg cell protein L^-1^ in the absence of CO, but the presence of CO in the headspace significantly increased growth to ∼120 mg cell protein L^-1^ (**Figure 2**). In addition, there was almost an order of magnitude increase in the amount of H_2_ produced compared to when CO was not present (**Figure 2**). At the end of growth, about 90% of the CO that was oxidized could be accounted for by the amount of H_2_ produced (after correcting for the H_2_ produced by the native *P. furiosus* Mbh in the absence of CO). This is close to the expected 1:1 ratio of CO oxidized to H_2_ produced.

Surprisingly, in the presence of CO, the Codh strain of *P. furiosus* was able to metabolize the acetate that it produced from the initial fermentation of the added sugar. We presume that this acetate is used for biosynthesis. As shown in **Figure 2**, in the absence of CO, the Codh strain produced approximately 3.5 mM acetate, but when CO was present, acetate production peaked at 1.3 mM at mid-log phase, just after CO began to be utilized. The acetate concentration then decreased to <0.5 mM as the CO concentration decreased to almost zero. Hence, it appears that CO oxidation provides energy, not only for growth, but also for acetate utilization as a source of carbon for biosynthesis. The kinetics of CO utilization and H_2_ production in the *P. furiosus* Codh strain are similar to that seen with *T. onnurineus* (Supplementary Figure S1), although *T. onnurineus* does not seem to be able to reutilize the acetate that is produced in the early growth phase. The *P. furiosus* parental strain COM1c did not utilize CO during growth, nor was growth inhibited by the presence of CO (Supplementary Figure S1).

**FIGURE 3:**
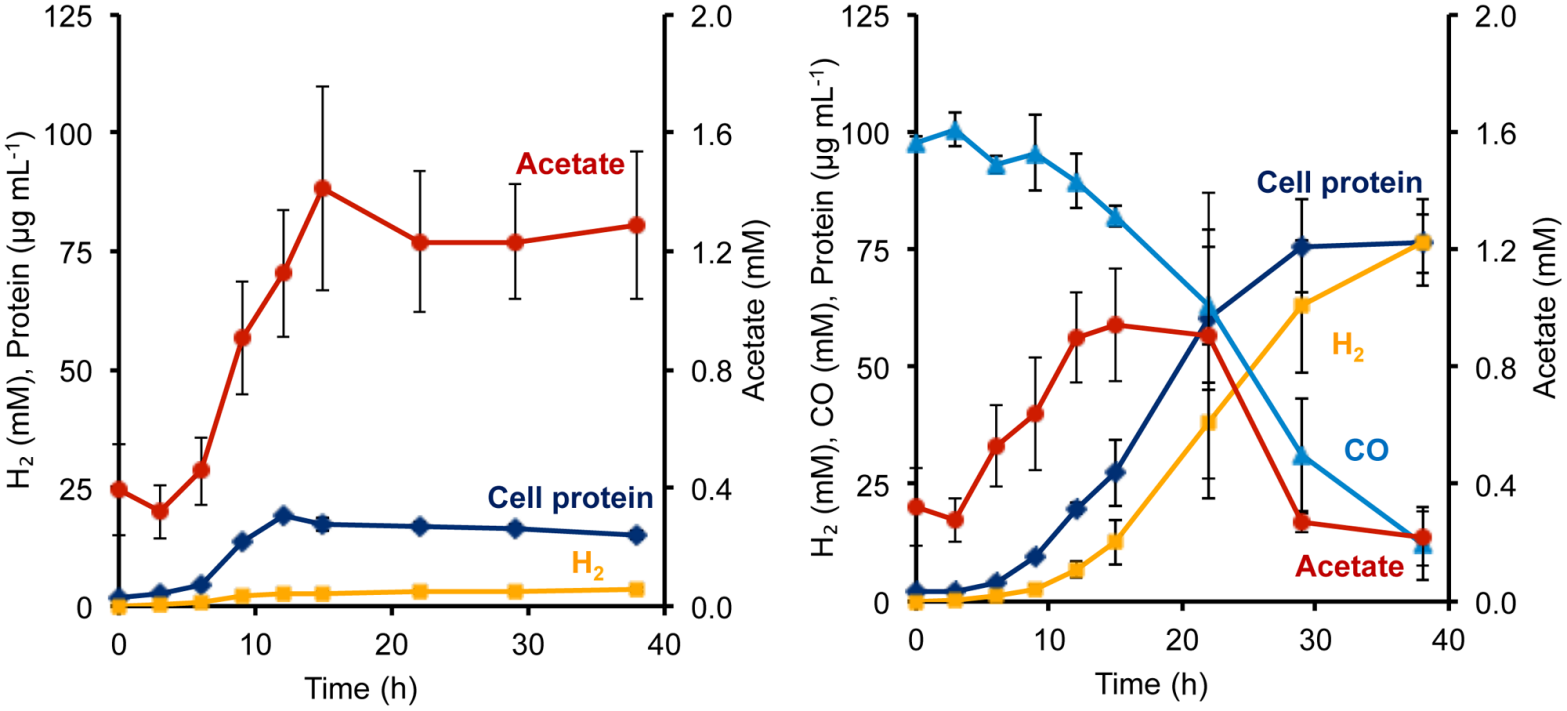
**Growth characteristics of *P. furiosus* strain Codh in the absence **(left)** and presence **(right)** of CO with limiting yeast extract (1 g L^-1^).** Compounds are represented as follows: blue triangles, CO; dark blue diamonds, cell protein; orange squares, H_2_; red circles, acetate. Error bars represent SD, *n* = 3.

During growth on sugar (maltose), *P. furiosus* generates reduced ferredoxin from sugar fermentation, and this is oxidized by the ferredoxin-dependent MBH to produce H_2_. During growth on peptides (1 g L^-1^ yeast extract), fermentation also generates NADPH, but its oxidation cannot be coupled directly to H_2_ production since MBH is specific for ferredoxin as the electron donor (Adams et al., 2001). Hence, in the absence of an external electron acceptor such as elemental sulfur, *P. furiosus* showed very poor growth on peptides, reaching <20 μg cell protein L^-1^, and only a low concentration of H_2_ was produced (3.6 mM; **Figure 3**). However, the addition of CO had a dramatic effect on the Codh strain of *P. furiosus* by significantly stimulating both growth and H_2_ production when peptides were used as the carbon source (**Figure 3**). Thus it is clear that the *P. furiosus* Codh strain obtains a significant amount of energy from the oxidation of CO. The contaminating glycans present in the yeast extract are assumed to be the source of the acetate that accumulated, and again, in the presence of CO, this was taken up and presumably assimilated into biomass (**Figure 3**). In addition to acetate, low concentrations of isobutyrate (up to 100 μM) and isovalerate (up to 200 μM) could also be detected in the medium at mid-log phase growth, and these are the products of amino acid fermentation. In the presence of CO, these organic acids were also consumed (data not shown).

## Discussion

The ability to utilize CO in the form of syngas, which is a mixture of mainly CO and H_2_, is an attractive option for the microbial production of liquid biofuels such as alcohols (Bengelsdorf et al., 2013). Herein we show that the 16-gene cluster encoding the trimodular respiratory Codh complex (Mrp-Mbh-Codh) can be functionally transferred between Thermococcales species. With the acquisition of the *T. onnurineus* Mrp-Mbh-Codh gene cluster, the *P. furiosus* Codh strain can utilize CO as an energy source. *P. furiosus* is not capable of autotrophic growth (Fiala and Stetter, 1986; Sokolova et al., 2004), and so addition of organic carbon sources such as peptides (yeast extract) is required for growth under carboxydotrophic conditions. It is interesting that in the presence of CO, the Codh strain is capable of metabolizing exogenous acetate as well as organic acids derived from branched chain amino acids. Organic acid utilization is presumably accomplished via the reverse reaction of the ADP-dependent acetyl-CoA synthetase (ACS) group of enzymes. These catalyze the ATP-dependent conversion of organic acids to the corresponding CoA derivative, and these can then be converted to amino acids for biosynthesis (Mai and Adams, 1996). In the case of strain Codh, the ATP is supplied by *P. furiosus* ATP synthase using the Na^+^ gradient generated by Codh during CO oxidation. The genome of *P. furiosus* encodes 10 different ACS isozymes that together are capable of reversibly converting acyl and aryl-CoAs derived from almost all of the amino acids to their corresponding organic acids (Scott et al., 2014).

The Codh gene cluster therefore could provide a metabolic advantage for microorganisms in hydrothermal vent systems where CO can be generated by volcanic action, although measured concentrations of CO in these environments are rather low (1–2 μM) (Cadle, 1980; Charlou et al., 2002). The exchange of CO utilization capabilities between Thermococcales species has a distinct advantage for the organisms involved when CO becomes available. In addition, organisms containing an Mrp-Mbh-Codh complex can be utilized to produce biohydrogen from waste gasses that contain CO (Kim et al., 2013), and the Codh strain of *P. furiosus* now has application for this process as well. Previously we have engineered *P. furiosus* to convert organic acids to the corresponding alcohols. Here we also have shown that in the Codh complex can be utilized to provide low potential electrons directly from CO to drive reduction of organic acids to the corresponding alcohols rather than generating hydrogen gas (Basen et al., 2014).

Using the CO-dependent formation of H_2_ as the *in vitro* assay for the Codh complex, it was shown that this activity is, as expected, strictly associated with the membranes in *P. furiosus* and in *T. onnurineus.* The Mbh and Mrp modules must be membrane-associated if the free energy from CO oxidation is to be conserved via the generation of an ion gradient. Surprisingly, the Codh module of the Codh complex, as measured by CO oxidation coupled to the reduction of an artificial dye (benzyl viologen), was mainly located in the cytoplasmic fractions of cells from both species. It is not clear if the dissociation of the Codh module from the membrane is a result of cell breakage, if it is naturally loosely associated with the membrane, or even if it is naturally soluble and transfers electrons to the Mrp-Mbh modules via a cytoplasmic ferredoxin. The latter possibility is not unreasonable as ferredoxin is the natural electron donor to the bimodular MBH complex (Mrp-Mbh) of *P. furiosus* (Sapra et al., 2003). A soluble Codh module is also consistent with the observation of high dye-linked CO-oxidizing activity in the cytoplasmic fractions of other anaerobic microorganisms with analogous CO oxidizing systems, such as *C. hydrogenoformans* and *Rhodospirillum rubrum* (Svetlitchnyi et al., 2001; Singer et al., 2006). However, in the case of *C. hydrogenoformans*, an eight-subunit membrane bound Codh-Ech complex was successfully purified (Soboh et al., 2002).

**FIGURE 4:**
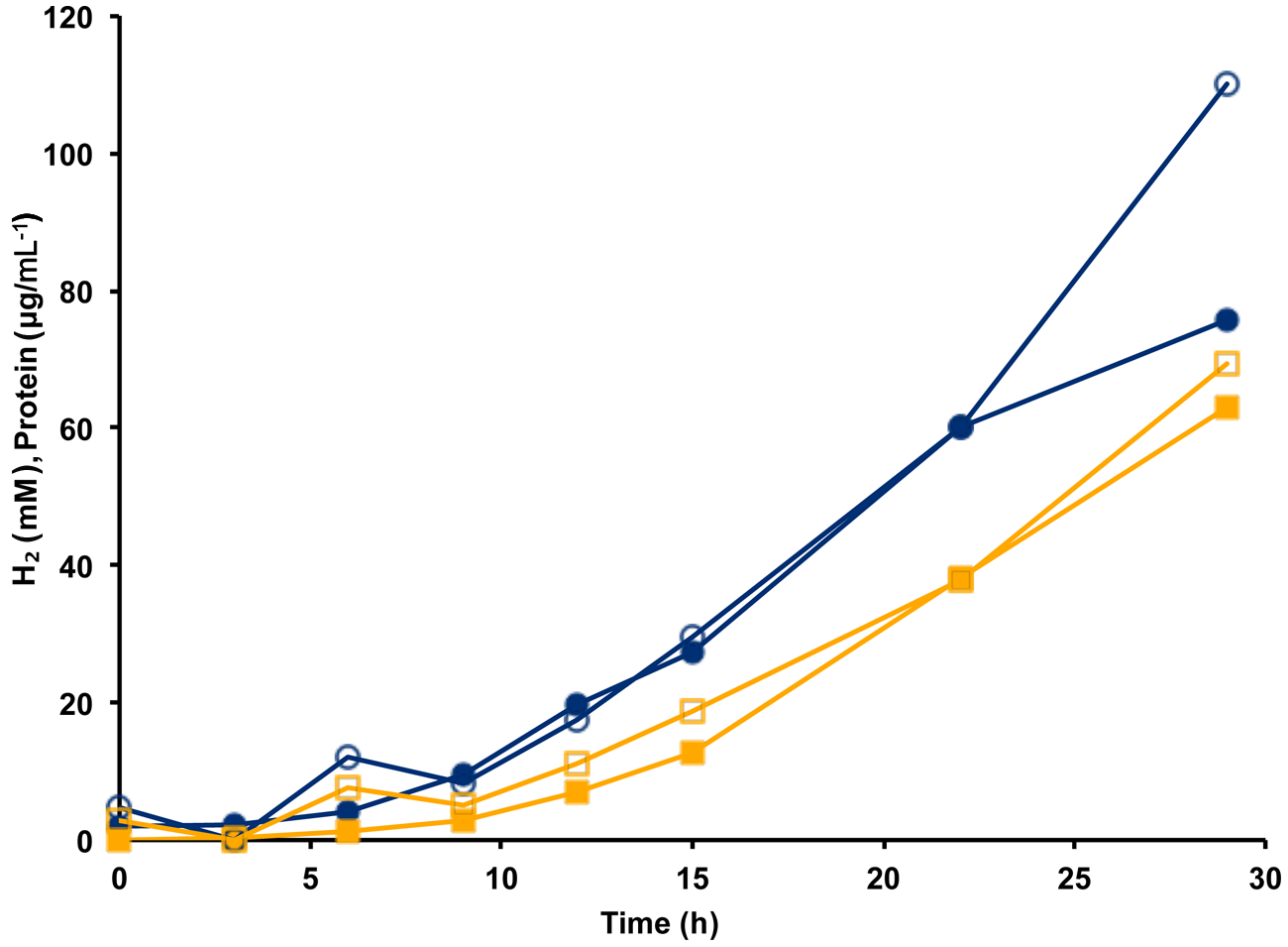
**Calculation of cell yield from CO utilization in the presence of minimal fixed carbon (1 g L^-1^ yeast extract) using an estimated yield of 0.3 ATP per mol CO oxidized/H_2_ produced.** Cell growth as represented by cell protein is indicated by blue lines with closed circles showing measured protein and open circles indicating calculated protein. Hydrogen formation is represented by orange lines with closed squares showing measured H_2_ (mM) and open squares indicating estimated H_2_ calculated from the amount of CO utilized.

The MBH complex consisting of two modules (Mrp-Mbh) is found in all known members of the Thermococcales order (Schut et al., 2013). The energy yield for *P. furiosus* MBH was estimated to be 0.3 ATP per mole of H_2_ produced (Sapra et al., 2003). Given that the Codh (Mrp-Mbh-Codh) complex contains the same two modules as MBH (Mrp-Mbh), we assume that the energy yields of the two systems are comparable and that 0.3 ATP is generated for every mole of CO oxidized. Based on the standard molar growth yield per mole of ATP (Stouthamer and Bettenhausen, 1973), we plotted the calculated cell protein yield based on the amount of CO oxidized using the data from **Figure 3**. As shown in **Figure 4**, during exponential growth, the calculated growth curve is reasonably close to that measured, so the production of 0.3 ATP per CO oxidized is a good representation of the energetics of CO metabolism. Previously, the ion translocating trimodular formate hydrogen lyase (FHL; Mrp-Mbh-Fdh) of *T. onnurineus* was heterologously expressed in *P. furiosus* (Kim et al., 2010; Lipscomb et al., 2014). However, in contrast to the situation reported here with Codh, the recombinant *P. furiosus* strain did not display formate-dependent growth on peptides or limiting amounts of sugar, even though it converted high concentrations of formate (>50 mM) to H_2_ (Lipscomb et al., 2014). In contrast to *P. furiosus, T. onnurineus* was reported to use formate oxidation coupled to H_2_ production as a source of energy (Kim et al., 2010). The Gibbs free energy for formate to hydrogen conversion at 80°C was calculated to be -2.7 kJ/mol and +1.3 kJ/mol at 25°C, and this was used as the rationale for why formate oxidation is energetically favorable at elevated temperatures (Kim et al., 2010). However, how *T. onnurineus* maintains local low concentrations of H_2_ during growth on formate in order to allow enough energy for ion translocation is not clear (Lim et al., 2012). In contrast, in the case of CO conversion to H_2_, more than enough energy is available for a minimum biological quantum of 20 kJ/mol to translocate ions over the membrane and allow ultimately for the synthesis of ATP (Schink, 1997).

## Author Contributions

GL constructed the recombinant strains. GS and DN carried out the physiological, biochemical, and chemical analyses. RK and MA designed and oversaw the research. All authors contributed to writing and editing the manuscript.

## Conflict of Interest Statement

The authors declare that the research was conducted in the absence of any commercial or financial relationships that could be construed as a potential conflict of interest.
