# Supplementary material for: Heterologous Production of an Energy-Conserving Carbon Monoxide Dehydrogenase Complex in the Hyperthermophile Pyrococcus furiosus
Source: Front Microbiol. 2016 Jan 29;7:29. doi: 10.3389/fmicb.2016.00029 (PMC4731540; doi:10.3389/fmicb.2016.00029)
Supplement: Supplementary file 1 [file Data_Sheet_1.DOCX]

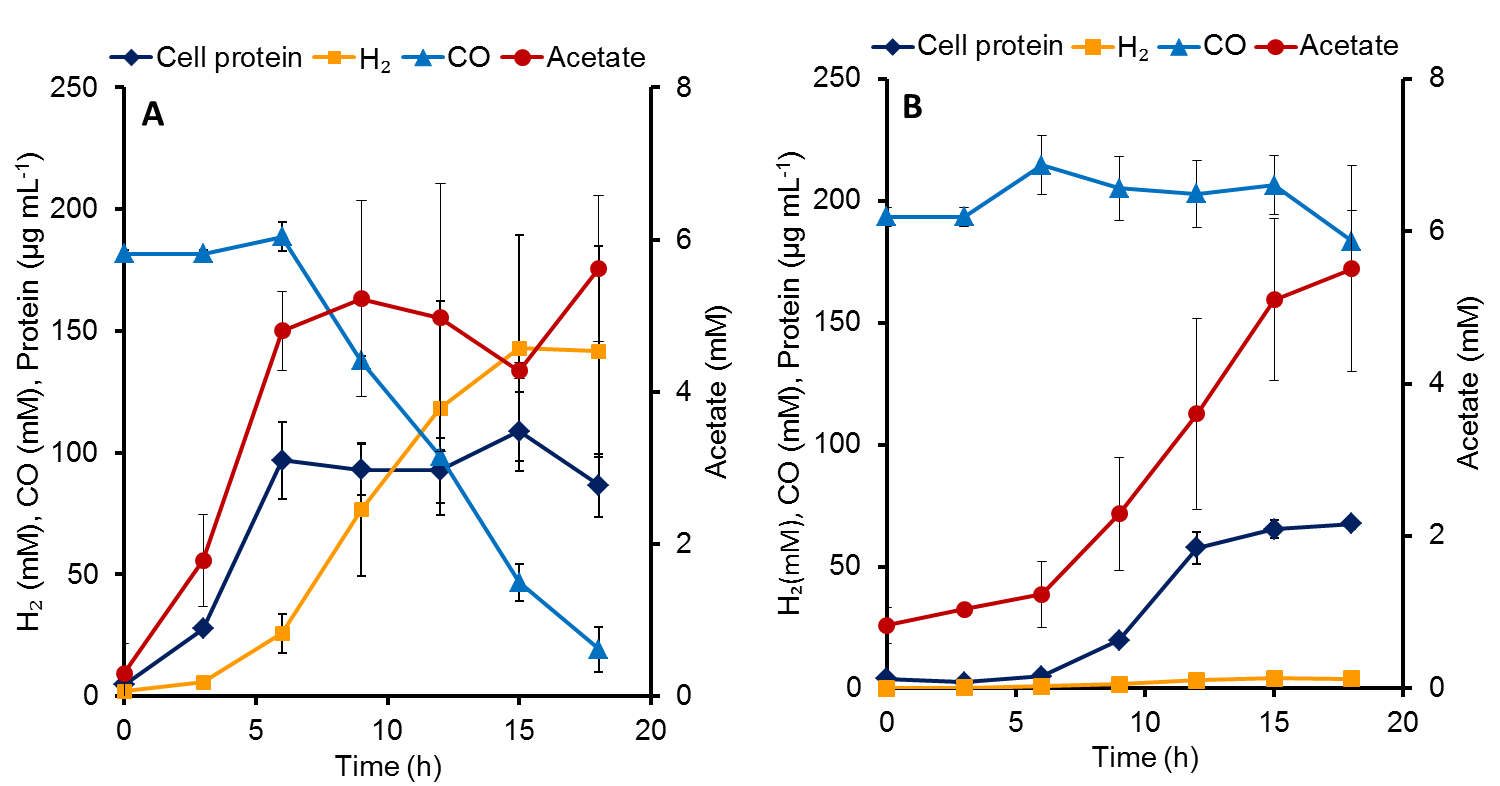


**FIGURE S1 |** CO-dependent growth characteristics of (**A**) *Thermococcus onnurineus* and (**B**) *Pyrococcus furiosus* parental strain COM1, with limiting maltose (0.5 g/l), and yeast extract(1 g/l).
